# In rats with estradiol valerate-induced polycystic ovary syndrome, the acute blockade of ovarian β-adrenoreceptors improve ovulation

**DOI:** 10.1186/s12958-019-0539-y

**Published:** 2019-11-19

**Authors:** Berenice Venegas, Lizzbeth Yureli De León Gordillo, Gabriela Rosas, Julieta A. Espinoza, Carolina Morán, Roberto Domínguez, Leticia Morales-Ledesma

**Affiliations:** 1 Biology of Reproduction Research Unit, Physiology of Reproduction Laboratory, Facultad de Estudios Superiores Zaragoza, UNAM, AP 9-020, CP, 15000 México, DF Mexico; 2 Área de Procesos Celulares Fundamentales, Facultad de Ciencias Biológicas, Benemérita Universidad Autónoma de Puebla, 72570 Puebla, CP Mexico; 3 Centro de Investigación en Fisicoquímica de Materiales, Instituto de Ciencias, Benemérita Universidad Autónoma de Puebla, Puebla, Mexico

**Keywords:** PCOS, β-Adrenergic receptors, Steroid hormones, Ovarian innervation, Ovulation

## Abstract

**Background:**

Polycystic ovary syndrome is characterized by hyperactivity of the ovarian sympathetic nervous system, increases in the content and release of norepinephrine, as well as decreases in the number of β-adrenoreceptors. In the present study, β-adrenoreceptors in the ovaries of rats with polycystic ovary syndrome were blocked and analyzed the resultant effects on ovulation, hormone secretion and the enzymes responsible for the synthesis of catecholamines.

**Methods:**

At 60 days of age, vehicle or estradiol valerate-treated rats were injected with propranolol [10^− 4^ M] into the ovarian bursas on oestrus day. The animals were sacrificed on the next day of oestrus, and the ovulation response, the steroid hormone levels in the serum and the immunoreactivity of tyrosine hydroxylase and dopamine β-hydroxylase in the ovaries were measured.

**Results:**

In animals with the induction of polycystic ovary syndrome and β-adrenoreceptor blocking, ovulation was restored in more than half of the animals and resulted in decreased hyperandrogenism with respect to the levels observed in the estradiol valerate-treated group. Tyrosine hydroxylase and dopamine β-hydroxylase were present in the theca cells of the growing follicles and the interstitial gland. Injection of propranolol restored the tyrosine hydroxylase and ovarian dopamine β-hydroxylase levels in rats with polycystic ovary syndrome induction.

**Conclusions:**

The results suggest that a single injection into the ovarian bursas of propranolol, a nonselective antagonist of β-adrenoreceptor receptors, decreases the serum testosterone concentration and the formation of ovarian cysts, improving the ovulation rate that accompanies lower levels of tyrosine hydroxylase and dopamine β-hydroxylase in the ovary.

## Background

Polycystic ovarian syndrome (PCOS) is the most common cause of infertility in women of reproductive age. It has a prevalence between 6 and 10% based on the U.S. National Institutes of Health criteria and 15% when the Rotterdam criteria are applied [1, 2]. PCOS is a multifactorial pathology that is characterized by hyperandrogenism, anovulation, presence of multiple ovarian cysts, irregularities in the menstrual cycle, and variable levels of gonadotropins [3, 4]. The etiology of PCOS is unknown, but intrinsic abnormalities in the synthesis and secretion of androgens are a probable basis for the syndrome [5]. Additionally, involvement of the sympathetic nervous system that innervates the ovaries during the development of the syndrome is suggested by studies in women with PCOS, where a high density of catecholaminergic nerve fibers has been shown [6]; further, in rats, the participation of sympathetic nerve fibers in the modulation of androgen secretion in the ovaries has been revealed [7], which may contribute to the etiology of PCOS [8]. In rats, the main catecholamine present in the ovaries is norepinephrine (NE), which stimulates steroidogenesis [9–11], follicular development [12–15] and ovulation [16–18] by regulating α- and β-adrenoreceptors (ADR) [19–21].

There is evidence that nonhormonal procedures result in PCOS. Luna et al., [22] showed that peripheral stimulation of β-adrenoreceptors (ADRB) with isoproterenol in wild-type adult rats promotes an increase in the number of precystic and cystic ovarian follicles without changes in plasma steroid levels, while blocking ADRB with propranolol in the same model inhibits their formation. The authors suggested that stimulation of ADRB activates the sympathetic nervous system of the rat ovary, which could be one mechanism of PCOS development and that they could be a therapeutic alternative for women with PCOS [22]. Fernandois et al. [23] showed that the prolonged blockade of β1 and β2-adrenoreceptors in 8- and 10-month rats, by i.p. daily injection of propranolol (5 mg/kg of body weight), during 60 days, recovered estrous cyclicity, elevated the ovulation rate, and levels of serum sexual steroids. We have previously shown that in the cyclic rat the acute blockade of β1 and β2-adrenoreceptors by propranolol injection at different days of the estrous cycle reduced the number of ova shed only in those animal treated on diestrus 2, without affecting ovulation in the other day of the cycle [24].

Several experimental models have been proposed to induce PCOS in neonatal, prepubertal or adult rats, depending on the phenotypic and physiological characteristics that are to be investigated, such as steroidal and nonsteroidal drugs (dehydroepiandrosterone, dihydrotestosterone, letrozole, and estradiol valerate (EV)-administration) [25–27] and genetic or environmental manipulations (genetically modified rat models as well as models developed with exposure to constant light or stress) [28, 29]. To study the relationship between the PCOS and sympathetic innervations, the most commonly used PCOS model is generated by a single injection of EV in prepubertal rats, which results in a polycystic ovary morphology, irregular estrous cycles [30, 31], alterations in basal and pulsatile luteinizing hormone (LH) concentrations and follicle stimulating hormone (FSH) concentrations and an increased androgen response to human chorionic gonadotropin stimulation [32]. The ovaries of rats injected with EV presented increased neural sympathetic activity [8, 32–34]. This increase is due to changes in the homeostasis of ovarian catecholamines that begin before the development of cysts and persist after their formation [8]. This change is accompanied by an increase in the release and content of NE from the nerve terminals to the ovary, an increase of tyrosine hydroxylase (TH) activity, the limiting enzyme for the synthesis of catecholamines, and a down regulation of ADRB2 in theca interstitial cells [8, 32, 35].

Previous studies have analyzed the participation of ovarian innervation in the development of PCOS in rats following EV injection, and increased activity of the sympathetic nerves of the ovary has been shown. The bilateral sectioning of the superior ovarian nerve (SON) in EV-treated rats restores ovulation [8], while the unilateral section of the SON in the same animal model restores ovulation mainly in the innervated ovary and the NE concentration was decreased only in denervated ovaries [36]. In a previous study [37] we showed that the elimination of noradrenergic fibers by guanethidine injection before the establishment of PCOS prevents the blockade of ovulation and hyperandrogenism. In animals with the PCOS peripheral sympathetic denervation by guanethidine also restores ovulatory capacity, but it was not as efficient in reducing hyperandrogenism. This suggests that the elimination of noradrenergic fibers before the establishment of PCOS prevents two characteristics of the syndrome: blocking of ovulation and hyperandrogenism [37]. Electroacupuncture treatment [33, 38] or voluntary exercise [39] in EV-treated rats reduces sympathetic activity, restores the oestrus cycle and ovulation, and normalizes LH secretion and steroidogenesis to regulate ADR.

Based on these evidences the aim of the present study was to analyze if a pharmacological acute blockade of ovarian ADRB restores ovarian functions in the EV model of PCOS.

## Materials and methods

### Animals

Newborn, female rats of the CII-ZV strain were kept with their mothers under controlled light conditions (lights on from 05:00 to 19:00 h) until weaning and were provided free access to food and water ad libitum under the same light conditions.

The animals were provided by the Facultad de Estudios Superiores-Zaragoza, UNAM, and the Bioethics Committee approved the experimental protocols. All procedures described in the present study were performed in accordance with the Guide for the Care and Use of Laboratory Animals of the Mexican Council for Animal Care (NOM-062-ZOO-1999) and to the Guidelines for the Use of Animals in Neuroscience Research from the Society for Neuroscience. Every effort was made to minimize the number of animals in each experimental group and to ensure minimal discomfort and pain.

### Experimental designs

Ten-day-old female rats were intramuscularly injected with 2.0 mg EV (Sigma Chemical Co., St. Louis, Mo. USA) that had been dissolved in 0.1 mL sesame oil. The vehicle group (Vh) was injected with a single 0.1 mL dose of sesame oil. Vaginal smear was performed daily after the vaginal opening was first observed.

At 60 days of age, the animals in vaginal oestrus were randomly assigned to one of the following four experimental groups:

1. *Vh group (n = 10).* Rats treated with sesame oil were sacrificed at 60 days of age, on oestrus day.
2. *Vh group plus propranolol (n = 10).* The ovarian bursas of rats treated with sesame oil were injected with 20 μL of propranolol [10^− 4^ M] (Sigma Chemical Co., USA) that was dissolved in 0.9% saline solution.
3. *EV group (n = 8).* Rats treated with EV were sacrificed at 60 days of age, on oestrus day.
4. *EV group plus propranolol (n = 9).* The ovarian bursas of rats treated with EV were injected with 20 μL of propranolol [10^− 4^ M] (Sigma Chemical Co., USA) that was dissolved in 0.9% saline solution.

### Surgery

Following the methodology previously described [40], each of the rats underwent a bilateral laparotomy under general anesthesia, and the ovaries were exteriorized to enable injection of 20 μL of propranolol into each one, with the aid of a Nano-Injector, Stepper Motorized (Stoelting Co, USA) and a 100 μL micro syringe (Hamilton, USA) equipped with a 29-gauge needle; the injection rate was 4 μL/min. To prevent fluid leakage, the needle was kept in the ovarian bursa for 2 min. Subsequently, the ovaries were carefully cleaned, dried, and returned to the abdominal cavity, and the skin and muscle were sutured. The surgeries were performed between 9:00 and 11:00 A.M.

### Autopsy procedures

Animals from each group were deeply anesthetized with pentobarbital between 9:00 and 11:00 A.M. following confirmation of oestrus by vaginal smear after the surgery. Blood was obtained by intracardiac puncture; it was allowed to clot and was centrifuged for 15 min. at 3000 RPM. The serum was stored at − 20 °C until progesterone, testosterone and oestradiol levels were measured. The animals were then perfused with a 200 mL of saline solution followed by 200 mL of 4% paraformaldehyde dissolved in a phosphate buffered solution (PBS). At autopsy, the oviducts were dissected, the number of ova shed was counted with the aid of a stereomicroscope, and ovulation was corroborated by observing the presence of corpora lutea (CL).

### Ovarian morphology

The ovaries were dissected and kept in paraformaldehyde for 24 h, rinsed with saline and kept in a PBS solution with 30% sucrose until histochemical processing. The paraformaldehyde-perfused ovaries were sectioned with a cryostat (Microm HM 525) at temperatures of − 20 °C, and the 10-μm thick section were subsequently mounted on coated glass slides. Ovarian serially sections of five animals from each group were stained with hematoxylin-eosin and examined under a light microscope. All sections from each group were analyzed for the presence of fresh CL and follicular cysts with a Leica binocular microscope (DM750) coupled to a Leica camera (ICC50 HD). The criteria used to define fresh CL were healthy cells with large nuclei and the presence of blood vessels. The follicular cyst structures were defined according to Brawer et al., [30].

### Immunofluorescence to TH and dopamine β-hydroxylase (DBH)

The ovarian sections of three animals taken at random from each experimental group (Vh, Vh + Pro, EV, and EV + Pro), were rinsed with PBS (pH 7.4) and were then rinsed twice with PBS with 0.5% Triton X-100. The nonspecific binding sites were blocked with IgG-free 2% bovine serum albumin (Sigma Chemical Co., USA). The sections were then incubated overnight at 4–8 °C with primary antibodies: polyclonal rabbit anti-TH antibody (1:200 sc-14,007 Santa Cruz Biotechnology Inc., USA) or polyclonal rabbit anti-DBH (1:200 sc- Santa Cruz Biotechnology Inc., USA), and the sections were subsequently incubated with a FITC-labelled goat anti-rabbit secondary antibody (Vector Labs Inc., USA). The slides were counterstained with Vectashield coupled with DAPI (Vector Labs Inc., USA) for nuclear staining. For negative controls, the primary antibody was substituted with PBS. Photomicrographs were taken using an Evolution VF Digital Camera (Media Cybernetics, Inc., USA) coupled with a fluorescence microscope (BX-41 Olympus Co.). From the ovarian sections of each animal, 10 ovarian follicles that exhibited the follicular antrum and the oocyte were selected, except in the cysts where the oocyte is absent (*n* = 3 animals per group with 10 pseudo-replicas per animal). Using the National Institutes of Health’s ImageJ software, the relative fluorescent to TH or DBH immunoreactivity was quantified following the methodology used previously [37, 40–42] . The color micrographs were converted to 8-bit grayscale images, the criteria used to define the intensity settings were constant between all sections (the selection area in square pixels were equal for each ovarian follicle analyzed). The regions of interest were randomly selected based on the visualization; the fluorescence intensity was quantified in a constant area of each class of follicle evaluated.

### Hormone measurements

The progesterone, testosterone and oestradiol serum levels were measured using a Radio-Immuno-Assay with kits purchased form Diagnostic Products (Los Angeles, CA). Progesterone results are expressed in ng/mL, and testosterone and oestradiol results are expressed in pg/mL. The intra- and interassay coefficients of variation were 8.35 and 9.45 for progesterone, 9.65 and 10.2 for testosterone, and 8.12 and 9.28 for oestradiol, respectively.

### Statistics

The results were expressed as the mean ± standard error (SE) for all experiments. The number of ova shed by ovulating rats was analyzed using Kruskal-Wallis tests, followed by a Mann-Whitney U-tests. The ovulation rate, expressed as the number of ovulating animals per number of treated animals, was analyzed using Fisher’s exact probability test. Hormone serum levels and immunoreactivity of TH or DBH were analyzed using one-way analysis of variance followed by Tukey test, with Graph Pad Software, Inc., (San Diego, CA, USA). A probability ≤5% was considered significant.

## Results

### Ovulation rate and number of ova shed (Table 1)

The animals injected with EV exhibited vaginal opening at 14 ± 0.0 days of age and were in oestrus according to the vaginal smear, which remained unchanged until the day of sacrifice. Animals injected with Vh exhibited vaginal opening at 35.1 ± 1.2 days of age and had 4-day oestrous cycles.

**Table 1:** Ovulatory response in rats with EV and blocking β-adrenergic receptors in ovaries at 60-day old

Groups	*N*	Ovulation rate	Ova shed
Vh	10	10/10	12.3 ± 0.7
Vh + Pro	10	10/10	8.4 ± 1.0^a^
EV	8	1/8^a,b^	13
EV + Pro	9	6/9^a,b,c^	4.0 ± 1.67^a^

Ovulation rate and number ova shed in animals treated with vehicle (Vh) or estradiol valerate (EV) at 10 day old and injected with propranolol (Pro) [10^−4^ M] [20 μL] into the both ovarian bursas at 60 day of age. The animals were sacrificed at next estrus day. Ovulation rate, ^a^*p* < 0.05 vs Vh group, ^b^*p* < 0.05 Vh + Pro or ^c^*p* < 0.05 vs EV group (Fisher’s test); number ova shed ^a^*p* < 0.05 vs Vh group (Kruskal-Wallis test)

In the Vh group, all the animals ovulated regardless of whether or not they were injected with propranolol. However, the number of ova shed was smaller in the Vh plus propranolol group than in the Vh group (Table 1).

In the EV group, 1/8 animals ovulated, while in the EV plus propranolol group, 6/9 of the microinjected animals ovulated. The number of ova shed by the EV group microinjected with propranolol was smaller than the number observed in the Vh group (Table 1).

### Hormone serum levels

**Fig. 1:**
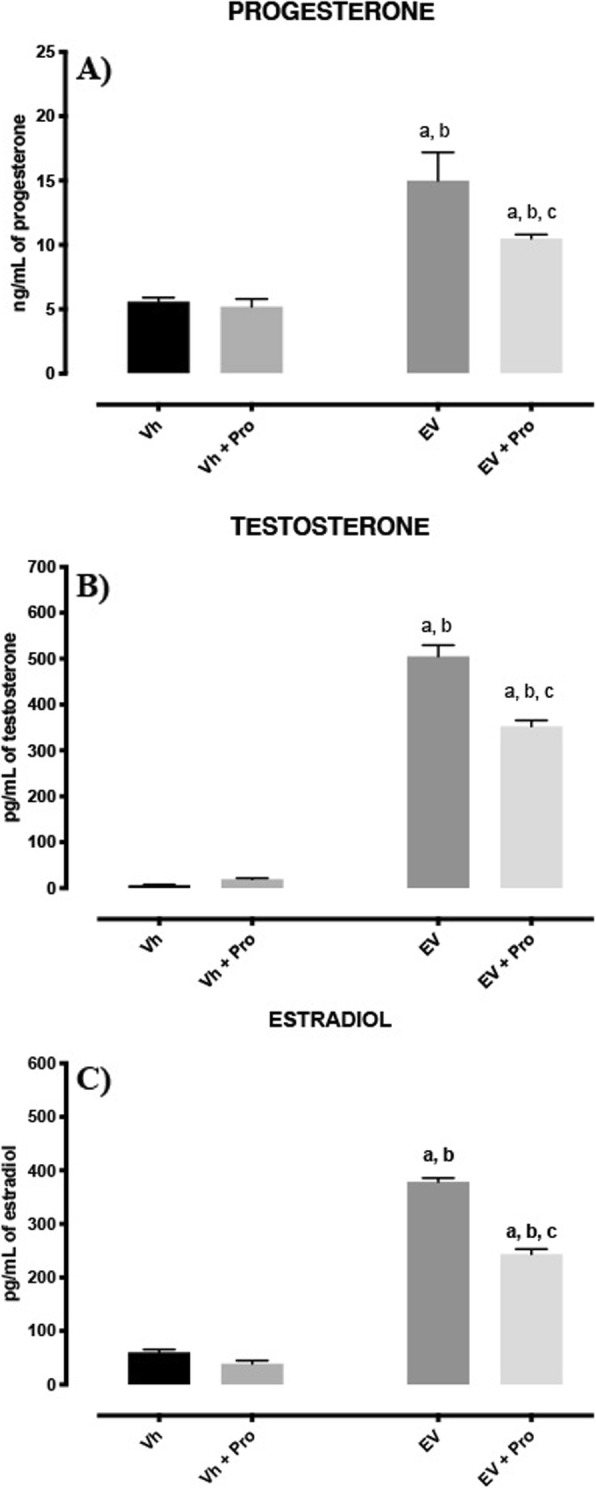
Mean ± SE of progesterone (**a**), testosterone (**b**) and estradiol (**c**) levels in the serum of rats with EV resulting from the blocking ovarian β-adrenergic receptors at 60-day old in oestrus day. The Vh- or EV-treated rats were injected with propranolol (Pro) [10^− 4^ M] in both ovarian bursas on oestrus day. Animals were sacrificed on the next oestrus day after surgery. ^a^*p* < 0.05 vs Vh group ^b^*p* < 0.05 vs. Vh + Pro group ^c^*p* < 0.05 vs. EV group (one-way analysis of variance, followed by Tukey test)

Microinjection of propranolol in both ovarian bursas of rats treated with Vh did not result in changed progesterone levels compared with the Vh group. Animals injected with EV exhibited higher concentrations of progesterone than the controls. The single injection of propranolol within the ovarian bursas in rats with EV resulted in lower progesterone levels than those observed in EV-injected rats (Fig. 1a).

In the Vh group, propranolol microinjection in both ovarian bursas did not modify testosterone levels compared with Vh-injected group. Testosterone levels in EV animals were higher than those in Vh-injected animals. In these animals, the microinjection of propranolol in both ovarian bursas resulted in lower testosterone levels than EV-treated group but higher testosterone levels than Vh-injected animals (Fig. 1b).

The microinjection of propranolol in Vh-treated animals did not change oestradiol levels compared with Vh-injected rats. The hormone levels in EV-treated animals were higher than those in Vh-treated animals. The microinjection of propranolol into both ovarian bursas resulted in lower oestradiol levels than EV-treatment group (Fig. 1c).

### Ovarian morphology

**Fig. 2:**
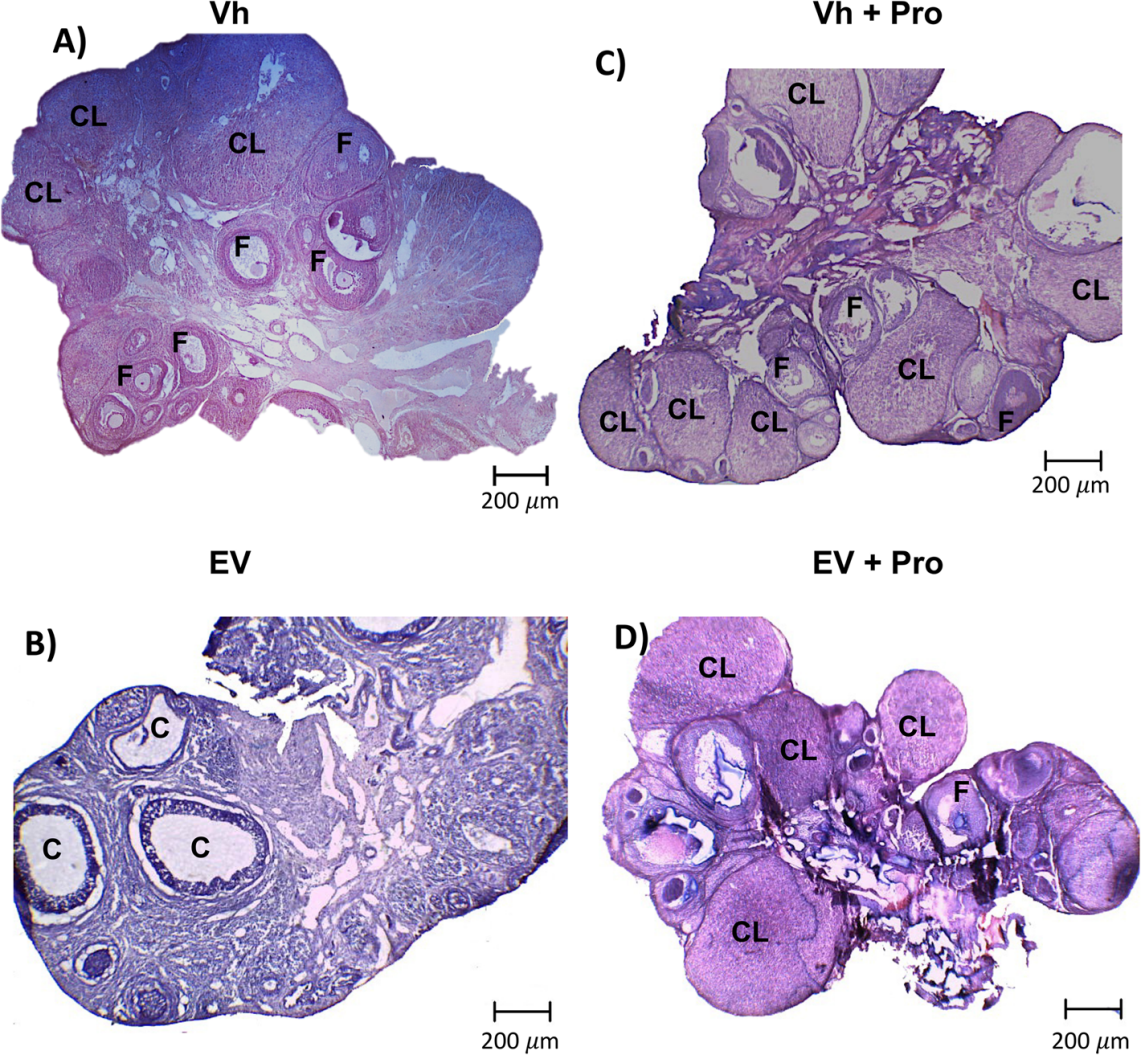
Ovarian morphology in EV-induced PCOS rats after blocking the ovarian β-adrenergic receptors at 60 days of age. Representative hematoxylin-eosin-stained 10 μm-thick sections showing the morphology of the **a** ovary from Vh-injected rats, **b** PCOS ovary from the EV group, **c**, **d** ovary from Vh- or EV-injected rats and Pro injection [10^− 4^ M] into the ovarian bursas at 60 days of age, sacrificed at 9:00 A.M. on the next day of oestrus. F: Follicle, C: Cyst, CL: Corpora Lutea. Bar 200 μm

The ovaries of rats injected with Vh and microinjected or not with propranolol in both ovarian bursas presented growing follicles at different stages and CL (Fig. 2a and c). The ovaries of rats injected with EV presented follicular cysts, and only the ovaries of a single rat had CL (Fig. 2b). In the ovaries of EV-treated rats that were microinjected with propranolol in both ovarian bursas (Fig. 2d), CL were observed as in the Vh group.

### TH and DBH immunoreactivity in ovarian tissue

**Fig. 3:**
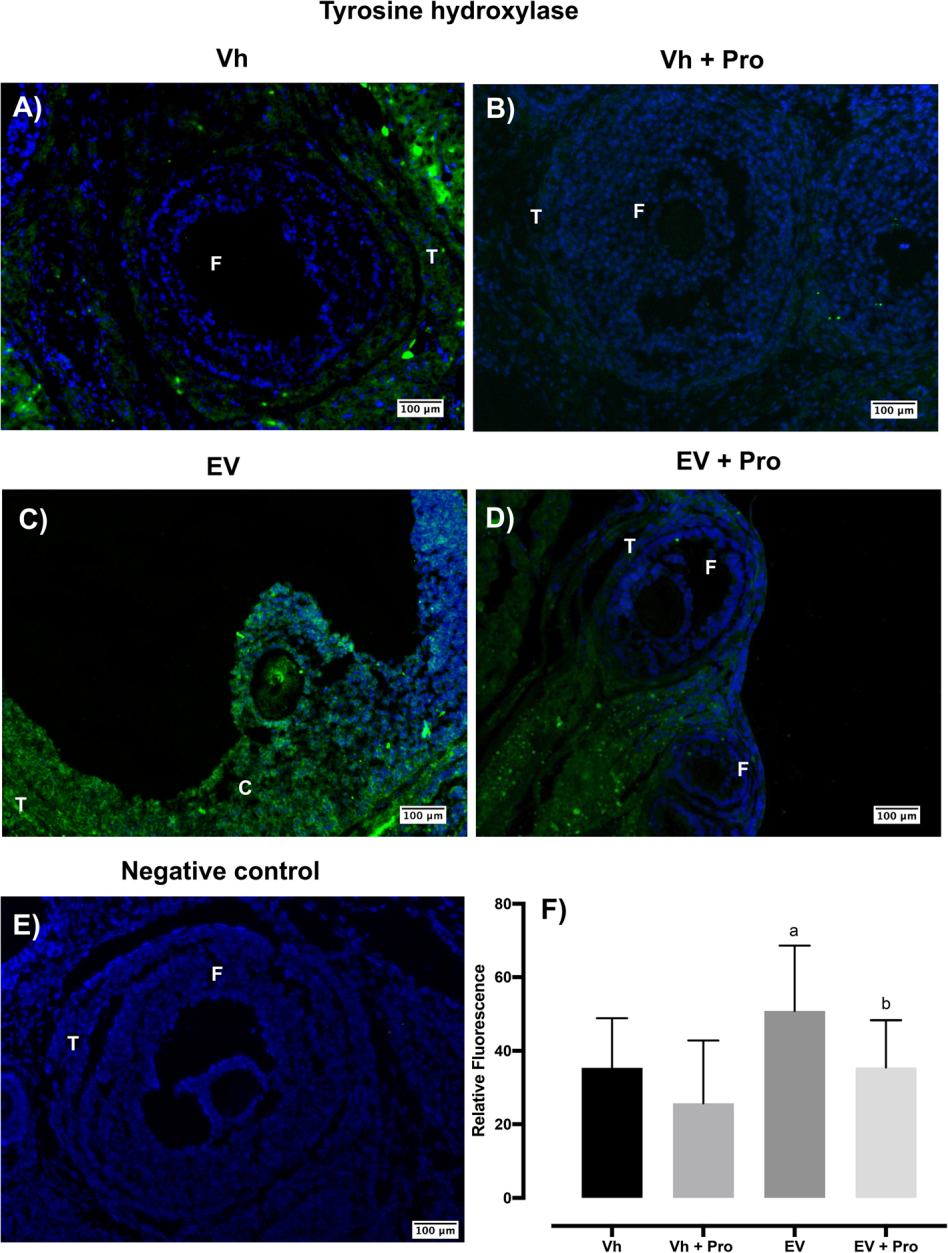
Distribution of TH in ovaries of Vh (**a**) or EV-treated rats (**c**) and before the bilateral injection of propranolol (Pro) (**b**, **d**) into the ovarian bursas. **e** Negative control where the primary antibody was substituted with PBS. The ovarian sections were stained with anti-TH antibody (green color), and nuclear staining was performed with DAPI (blue color). TH is observed throughout the ovary, including the F: follicle and T: Theca cell. Bar 100 μm. **f** ImageJ analysis of TH relative fluorescence means ± SE (*n* = 3 animals per group with 10 pseudo-replicas per animal), ^a^*p* < 0.05 vs Vh group; ^b^*p* < 0.05 vs. EV group (one-way analysis of variance, followed by Tukey)

The data had a normal distribution (TH fluorescence intensity of follicles with antrum: *p* value 0.9702 and cyst: p value 0.5176, Shapiro-Wilks normality test). TH and DBH immunoreactivity were found only in the interstitial tissue and theca cells of antral follicles. Compared to the Vh group, the TH immunoreactivity was not significantly different in the ovarian tissue of Vh- propranolol injection-treated rats. The highest intensity of TH immunoreactivity was observed in the theca cells of the ovarian follicles from the EV group. Propranolol injection into the ovarian bursas in EV-treated rats restored TH immunoreactivity, with respect to the EV group (Fig. 3).

**Fig. 4:**
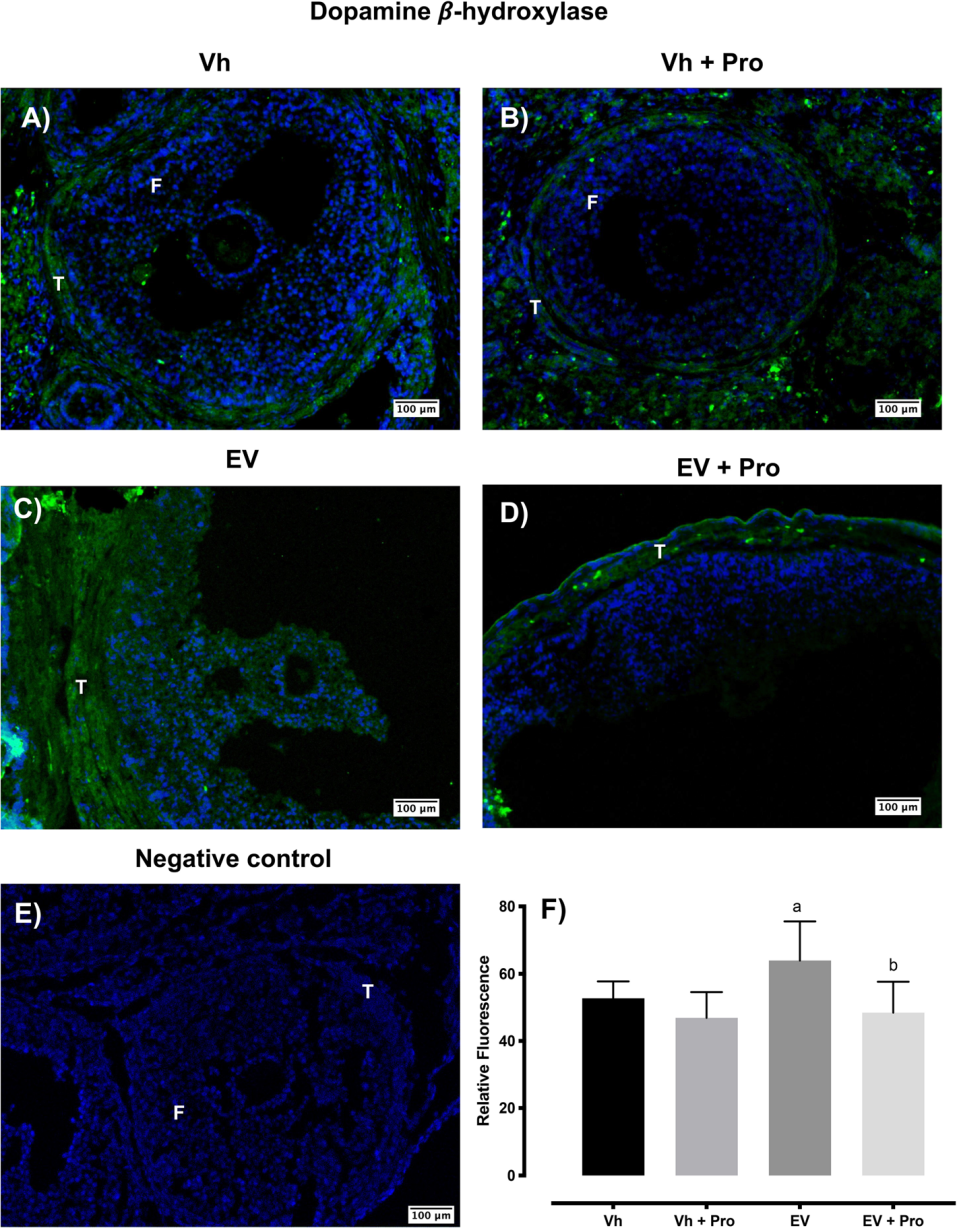
Distribution of DBH in ovaries of Vh (**a**) or EV-treated rats (**c**) and before the bilateral injection with propranolol (Pro) (**b**-**d**) into the ovarian bursas. The ovarian sections were stained with anti-DBH antibody (green color), and nuclear staining was performed with DAPI (blue color). **e** Negative control where the primary antibody was substituted with PBS. DBH is observed throughout the ovary, including the F: follicle, T: theca cell. Bar 100 μm. **f** ImageJ analysis of DBH relative fluorescence means ± SE (*n* = 3 animals per group with 10 pseudo-replicas per animal), ^a^*p* < 0.05 vs Vh group; ^b^*p* < 0.05 EV group (one-way analysis of variance, followed by Tukey)

The microinjection of propranolol did not modify the DBH immunoreactivity in the Vh group. The DBH immunoreactivity in the ovaries of rats injected with EV was higher with respect to the Vh group. Propranolol injection into the ovarian bursas of EV-treated rats restored DBH immunoreactivity in ovarian tissue with respect to the EV group (Fig. 4).

## Discussion

The results of the present study show that acute blocking of ADRB in ovaries with PCOS reestablishes ovulation in more than half of the animals, decreases progesterone, testosterone and oestradiol levels, prevents the development of ovarian cysts (as determined by the observation of ovarian tissue with growing follicles or presence of CL), and restores the enzymes responsible for the synthesis of NE to their basal levels.

Hyperactivity of the sympathetic ovarian system has been proposed to be associated with hyperandrogenemia [5, 7, 43, 44]; however this relation is not yet clear [43, 45]. Lara et al. [8] showed that the levels of NE in the ovaries increased slightly at 30 days after an EV injection. When the animals were analyzed 60 days after injection with EV, they had higher levels of ovarian NE and testosterone than the controls did. Rats injected with EV develop PCOS morphology, show a downregulation of ADRB2 and show an increase in the nerve growth factor (NGF) and its low affinity receptors in the ovary [7, 8, 32, 46]. This association suggests that NGF [7, 43, 44] induces androgen overproduction in ovaries with PCOS, which is also a result of hyperactivation of the catecholaminergic system on ovarian steroid-secreting cells [32]; however, when the NGF actions were blocked in the ovaries, the ovarian functions are restored [46].

Previous studies have shown that EV-treated rats with unilateral section of the SON restored ovulation by the innervated ovary and normalized the testosterone and oestradiol levels [36]. This result suggests that noradrenergic fibers arrive by SON participate in the hyperandrogenism in the PCOS model. On the other hand, Linares et al. [47] showed that the bilateral section of the vagus nerve (VG) in EV-injected rats restored ovulation in both ovaries, suggesting that the neural information carried by the SON and VG plays a role in the regulatory mechanisms of development and maintenance of PCOS.

Other studies using agonists and antagonists of ADR have suggested that α-adrenoreceptors (ADRA) and ADRB are present in the ovaries [10, 11, 19, 48–51]. Consistent with Ojeda and Lara [52] shown that NE acts on ADRB into theca and granulosa cells and stimulates the progesterone and testosterone secretion, but not oestradiol. Likewise, in EV-treated rats, the progesterone and androgen secretion increased in a NE-dependent manner [34].

According to Luna et al. [22], the ovaries of adult rats injected daily with isoproterenol for 10 days, on day 11 secreted a higher amount of androstenedione than the ovaries of the control group. Such increase was not observed in rats studied 30 days after isoproterenol treatment, besides ovarian cysts were still present, the adrenergic activity is similar to the control group, suggesting that after ending the treatment with isoproterenol, the animals began their recover. This response is different in EV-treated rats who have hyperandrogenism and hyperactivation noradrenergic for longer periods [8]. After 56 days of EV injection, several groups have described the presence of follicular cysts and ovarian noradrenergic activity remains higher than normal [8, 32, 34, 36, 46, 53]. Then, we suppose that the mechanisms involved in the formation of the polycystic ovary induced by isoproterenol and EV are different.

The findings from this study showed that a single propranolol injection into the ovaries of EV-treated rats improves the ovulation rate, as evidenced by the presence of CL. Moreover, the progesterone and testosterone levels were lower in EV-treated rats and microinjected with propranolol than those treated only with EV; hence, the ADRB blocker begins to restore ovarian steroidogenesis. We suggest that if the blockage of the ADRB receptors is maintained, the concentration of steroid hormones could decrease even more. Although not all rats in the EV group plus propranolol ovulated, there was a decrease in testosterone concentration in all animals treated with the ADRB receptor antagonist, which suggests variability in animals. It has been suggested that in prepubertal animals, the regulation of enzymes that participate in progesterone, testosterone and oestradiol synthesis does not occur in parallel. This suggests that the mechanisms of regulating the synthesis of each hormone are not regulated by the same signals and that the changes in the steroid hormone levels are not explained by the changes in gonadotropin secretion [54].

According to Fernandois et al., [23] there exists a correlation between reproductive aging and PCOS; both processes are accompanied by increased intraovarian sympathetic tone. In their study, it was proposed that after 2 months of blocking the ADRB, there was a reactivation of follicular development, an improved ovary cycling activity, an increased ovulation rate and a decrease in the number of cystic structures. Luna et al., [22] proposed that PCOS could be induced by ADRB activation in rats and could be prevented by simultaneous administration of an agonist and an antagonist of ADRB. In the present study, a single propranolol injection into the ovarian bursas of EV-rats showed ovarian morphology with follicular development and the presence of CL, indicating that the animals ovulated. However, this treatment was not able to reestablish ovarian functions in all animals. Espinoza et al. [37] showed that chronic administration of guanethidine (a drug that destroys noradrenergic fibers), prior to the induction of PCOS with EV, prevents the blockage of ovulation and hyperandrogenism. However, animals that have already developed PCOS are not able to reduce testosterone levels; despite pharmacological denervation, neural signals arrive in the ovaries via the SON.

It is possible that when ADRB are blocked, NE acts on α-adrenoreceptors, maintaining high testosterone levels, despite treatment with propranolol. Manni et al., [38] showed that the expression of ADRA1 was higher in the ovaries of rats with PCOS. Although the effect of the ADRA activation on ovarian steroidogenesis in PCOS rats has not been studied, it has been shown that in cultured granulosa cells obtained from adult rats, phenylephrine (an ADRA1A agonist) stimulates the secretion of progesterone [11], which is a precursor of testosterone.

According to Morales-Ledesma et al. [36] the NE release in EV-treated rats increased from the sympathetic fibers to ovaries. This change is associated with higher TH activity [8, 32, 35]. In the present study, we show that TH and DBH immunoreactivity is present in the theca-interstitial cells of EV-treated rats, and this activity is likely associated with the synthesis and secretion of testosterone. To our knowledge, this study is the first to show that a single propranolol injection into the ovarian bursas in EV-treated rats decreases TH immunoreactivity. These observations suggest that the functional activity of ovarian sympathetic tone is diminished by blocking ADRB. Likewise, DBH immunoreactivity is decreased in EV-treated rats. This finding suggests that the increase in TH activity produces a downregulation of DBH immunoreactivity in the ovaries as a way of producing negative feedback of NE synthesis.

## Conclusions

The results suggest that acute ovarian blocking of ADRB in animals with EV-induced PCOS improves the ovulation rate, decreases the testosterone levels and promotes follicular development by decreasing the hyperactivity of the ovarian noradrenergic system.

## Data Availability

The datasets generated during and/or analyzed during the current study are available from the corresponding author on reasonable request.

## Abbreviations

ADR: Adrenoreceptors
ADRA: α-adrenoreceptor
ADRB: β-adrenoreceptor
CL: Corpora lutea
DBH: Dopamine β-hydroxylase
EV: Oestradiol valerate
FSH: Follicle stimulating hormone
LH: Luteinizing hormone
NE: Norepinephrine
NGF: Nerve growth factor
PBS: Phosphate buffer solution
PCOS: Polycystic ovarian syndrome
SON: Superior ovarian nerve
TH: Tyrosine hydroxylase
Vh: Vehicle
VN: Vagus nerve
